# Older Adults Benefit from Symmetry, but Not Semantic Availability, in Visual Working Memory

**DOI:** 10.3389/fpsyg.2017.02373

**Published:** 2018-01-24

**Authors:** Colin J. Hamilton, Louise A. Brown, Clelia Rossi-Arnaud

**Affiliations:** ^1^Department of Psychology, Northumbria University, Newcastle upon Tyne, United Kingdom; ^2^School of Psychological Sciences and Health, University of Strathclyde, Glasgow, United Kingdom; ^3^Department of Psychology, Sapienza Università di Roma, Rome, Italy

**Keywords:** visual working memory, aging, functional architecture, symmetry, semantic affordance

## Abstract

Visual working memory exhibits age effects that are amongst the largest observed in the cognitive aging literature. In this research we investigated whether or not older adults can benefit from visual symmetry and semantic availability, as young adults typically do. Visual matrix pattern tasks varied in terms of the perceptual factor of symmetry (Experiment 1), as well as the availability of visual semantics, or long-term memory (LTM; Experiment 2). In Experiment 1, within a visual memory span protocol, four matrix pattern sets were employed with discrete symmetry characteristics; random, vertical, horizontal, and diagonal symmetry. Encoding time was 3 s with a 2 s maintenance interval. The findings indicated a significant difference in span level across age groups for all of the symmetry variants. More importantly, both younger and older adults could take advantage of symmetry in the matrix array in order to significantly improve task performance. In Experiment 2, two visual matrix task sets were used, with visual arrays of either low or high semantic availability (i.e., they contained stimuli with recognizable shapes that allow for LTM support). Encoding duration was 3 s with a 1 s retention interval. Here, the older adult sample was significantly impaired in span performance with both variants of the task. However, only the younger adult participants could take advantage of visual semantics. These findings show that, in the context of overall impairment in individual task performance, older adults remain capable of employing the perceptual cue of symmetry in order to improve visual working memory task performance. However, they appear less able, within this protocol, to recruit visual semantics in order to scaffold performance.

## Introduction

Visual Working Memory (VWM) is the ability to maintain and process visual details, such as patterns, orientations, and colors, over the short term (i.e., periods of seconds). There is substantial evidence to indicate that VWM performance demonstrates significant age associated deficits (Smith et al., 1990; Bruyer and Scailquin, 1999; Leonards et al., 2002; Beigneux et al., 2007; Logie and Maylor, 2009; Johnson et al., 2010; Swanson, 2017). It is not yet known precisely why visual working memory is particularly age-sensitive, although researchers have recently suggested that older adults' VWM may have the same capacity as younger adults, but with less precision (Peich et al., 2013; Ko et al., 2014). It has also been shown that processing speed contributes to older adults' VWM capacity (Brown et al., 2012), particularly when there are multiple objects to be encoded, retained, and recalled (Guest et al., 2015). The aim of this research was to investigate the extent to which the perceptual and semantic properties of visual stimuli can influence VWM task performance across younger and older adult age groups. Performance was compared on experimental variants of two previously validated quantitative, capacity-based measures of VWM, in order to provide further insight into *where* and *why* there are age-associated changes in VWM.

Multiple resource accounts of working memory, such as those by Baddeley (2012) and Logie (2011), have made explicit the importance of domain-specific verbal and visuo-spatial slave systems, which work in conjunction with relatively amodal executive attentional resources. These working memory sub-systems can also interact with long-term memory to take advantage of stored knowledge. The notion of a functional architecture (Hamilton et al., 2003; Hamilton, 2011), in which a range of mechanisms underlie VWM task performance, raises important questions regarding the mechanism/s responsible for age associated changes in performance. One idea is that the observed age change results from a common global change in cognitive processing (Baltes and Lindenberger, 1997), such as processing speed (e.g., Salthouse, 1996). However, while the “common cause” hypothesis can account for a proportion of the variance in age-related cognitive decline, it is likely that domain-specific changes are also required to be able to provide a more complete explanation of the mechanisms underlying cognitive aging (e.g., Lindenberger and Ghisletta, 2009). Specifically, there may be differential age-related changes in the availability of specialized cognitive resources relevant to the task at hand. In the case of visual working memory, this would include short-term visual storage and/or related mechanisms, such as executive attentional resources and temporary activation of visual semantics (Logie, 2011).

Research has shown that VWM may be particularly susceptible to age-related degeneration, with potential benefit from scaffolding by more generic, executive cognitive functioning (e.g., Park and Reuter-Lorenz, 2009; Reuter-Lorenz and Park, 2010). Indeed, the evidence suggests that working memory task performance may vary quite idiosyncratically. For example, visual matrix tasks which involve recalling an abstract black and white cell matrix stimulus, with 50% black, and 50% white cells, such as the Visual Patterns Test (VPT; Della Sala et al., 1997, 1999), demonstrated steeper linear declines across the adult lifespan than other working memory tasks (Logie and Maylor, 2009; see also Johnson et al., 2010). These included other processing-intensive working memory tasks such as a sentence verification measure of verbal working memory span, a test of prospective memory, and a visual memory task requiring binding of color, shape, and location features (Allen et al., 2013). Visual matrix tasks, like other higher-order, complex WM tasks, could be considered to exemplify the problem in identifying what specific processes change with age, as the task is likely to involve both domain-specific maintenance and domain-general executive resources (Cowan, 1988, 2005, 2016; Hamilton et al., 2003). A multiple resource account could readily ascribe age-related VPT performance to the change in efficacy of a domain-specific process such as the *visuo-spatial sketch pad* (VSSP, Baddeley, 2012) or a *visual cache* process (Logie, 2011); the specialized visual storage mechanisms in these respective multiple component models of working memory. However, additionally, there is consistent evidence that in children and young adults the task demands are also associated with the recruitment of domain-general executive resources (Hamilton et al., 2003; Brown et al., 2006; Rudkin et al., 2007; Brown and Wesley, 2013).

Thus, differences between younger and older adults in VPT performance could be derived from age-related challenges specifically to temporary visual storage, and/or to broader working memory processes such as domain-general executive attention. To explore the involvement of broader working memory mechanisms to visual working memory performance in young adults, Brown and Wesley (2013) employed two VPT stimulus sets which varied in the extent to which the patterns could be verbalized. Brown et al. (2006) previously established that the high verbalizable set led to a greater VPT task performance in younger adults. Brown and Wesley showed that secondary task random tapping during the maintenance interval removed this advantage. Crucially, neither a manual, non-executive-demanding spatial tapping task, nor articulatory suppression for limiting repetition of verbal codes, removed the advantage associated with more verbalizable stimuli. Thus, the random interval tapping interfered specifically with the available executive attentional resources, which could ordinarily be used to access and retrieve semantic and/or verbal codes from LTM (Craik and Byrd, 1982; Logie, 2016) and to integrate them with the novel VPT patterns (see also Hamilton et al., 2003; Verhaeghen et al., 2006; Ricker et al., 2010). Brown and Wesley concluded, therefore, that there is a cognitive cost associated with strategically retrieving meaning and associating it with the otherwise abstract visual material. Thus, the executive demand could therefore underlie some of the age-related variance in VWM.

However, Sun et al. (2011) pointed to yet another potential explanation for age-related changes in VWM task performance. They distinguished between two characteristics of the stimuli which could contribute to task performance. The first was the notion of *perceived complexity* which was dependent upon the participants' expertise or familiarity with the stimuli. Whilst in principle the VPT protocol employs a novel pattern, as noted above, it is clear that typical young adults will strive to employ other cognitive resources to the task, such as by verbalizing, or extracting meaning from, patterns or pattern components (Brown et al., 2006; Brown and Wesley, 2013). However, a second construct was *physical complexity*, which in a VPT stimulus could refer to the proximity, continuity, or symmetry characteristics of the black cells (Attneave, 1957; Chipman, 1977).

The contribution of physical complexity characteristics to VWM task performance therefore identifies another process which could contribute to the age associated changes observed in VWM which, we understand, is yet to be specifically addressed with visual matrix tasks in an older adult sample. Structure within the to-be-remembered pattern will afford the opportunity for redundancy, enabling local elements of the pattern to be predictable from more global characteristics (e.g., Pieroni et al., 2011; Brady and Alvarez, 2015; Kaiser et al., 2015; Gao et al., 2016). Previous research has focused upon the physical characteristics associated with Gestalt properties of proximity, continuity, symmetry, etc. (e.g., Jiang et al., 2000; Woodman et al., 2003; Rossi-Arnaud et al., 2006, 2012; Pieroni et al., 2011).

The research of Rossi-Arnaud and colleagues has systematically investigated the contribution of symmetry in the pattern array within a context of sequential and simultaneous presentation formats. In their matrix pattern protocol, an increasing number of red cells were superimposed upon a 5 × 5 array of black cells. In young adult samples, within a simultaneous presentation format, arrays possessing vertical, horizontal, or diagonal symmetry were more effectively recalled than random arrays. In contrast, within a sequential presentation context, only arrays with vertical symmetry showed an advantage over a random pattern. Critically, this advantage of symmetry in simultaneous presentation contexts was not dependent upon the employment of executive attention. This lack of executive demand was demonstrated with the use of the dual task paradigm using a task switching secondary task (Rossi-Arnaud et al., 2006; Pieroni et al., 2011). This suggested that the encoding of symmetry into visual working memory was relatively “automatic,” or cost-free, as for the encoding of feature binding in young adults (Allen et al., 2006; Baddeley et al., 2011).

Consequently, in a visual matrix-type task, age-related differences may be due to deficits in either issues associated with the perceptual complexity of the pattern or executive attentional resources required for retrieval of LTM semantics, or some combination of both. One of the major accounts of cognitive differences associated with young and older adults has suggested that there are decreasing attentional resources available in older adulthood (e.g., May et al., 1999; Phillips and Hamilton, 2001; Braver and West, 2008; Healey and Kahana, 2016). Healey and Kahana further suggested that a key process was the ability to employ richly detailed context from LTM in order to facilitate the retrieval of the memorandum, which was compromised in adult aging. Thus, if younger adults typically draw upon semantics in visual matrix task performance (Brown and Wesley, 2013), and this process is compromised in older adults, then this could contribute to the effects of age in VWM capacity, as measured by a visual matrix task. Specifically regarding the contribution of bottom-up perceptual cues such as pattern symmetry, evidence suggests that adult aging is negatively associated with changes in visual function (e.g., Roudaia et al., 2008) and this has also been observed in the context of symmetry detection (Herbert et al., 2002). However, the extent to which a perceptual process such as symmetry compromises a higher level cognitive task such as visual working memory is an ongoing debate in the aging literature (see La Fleur and Salthouse, 2014; Houston et al., 2016). The primary aim of the current research was therefore to examine the effects of aging, visual symmetry, and semantic coding to visual working memory task performance, in order to understand the extent to which low-level perceptual processes, and higher-level strategic, executively-demanding processes contribute to VWM performance in younger and older adults.

## Experiment 1

In the first study, younger and older adults carried out a visual matrix symmetry task in which the patterns varied in their symmetry properties. The patterns were either random, or vertically, horizontally, or diagonally symmetrical. It was predicted that, given the small decrement in symmetry detection associated with age (Herbert et al., 2002), then there would be some reduction in the efficacy in which older adults take advantage of symmetry in the array pattern.

### Methods

#### Design

The experiment took the form of a cross sectional mixed factorial 2 × 4 design, and investigated the effects of adult age group (younger, older) and symmetry (control: random symmetry, vertical symmetry, horizontal symmetry, diagonal symmetry; repeated measures) on VWM capacity, as measured by the span level achieved in each task condition.

#### Participants

The sample comprised 50 participants in total. There were 20 younger adults, who were opportunistically sampled from the Department of Psychology, Sapienza, University of Rome. This group had a mean age of 23.85 (*SD* = 1.90; min = 20, max = 27) years, and 7 were female. There were 30 older adults, drawn from the North East Age Research cohort in the North East of England (Rabbitt et al., 2004). The group had a mean age of 81.66 (*SD* = 5.69; min = 73, max = 93) years, and 22 were female. This group were all living independently in the community. This study was carried out in accordance with the recommendations of Committees for Ethics, Department of Psychology La Sapienza, and Department of Psychology, Northumbria University with written informed consent from all subjects. All subjects gave written informed consent in accordance with the Declaration of Helsinki. The protocol was approved by the two ethics committees identified above.

#### Materials and procedure

**Figure 1:**
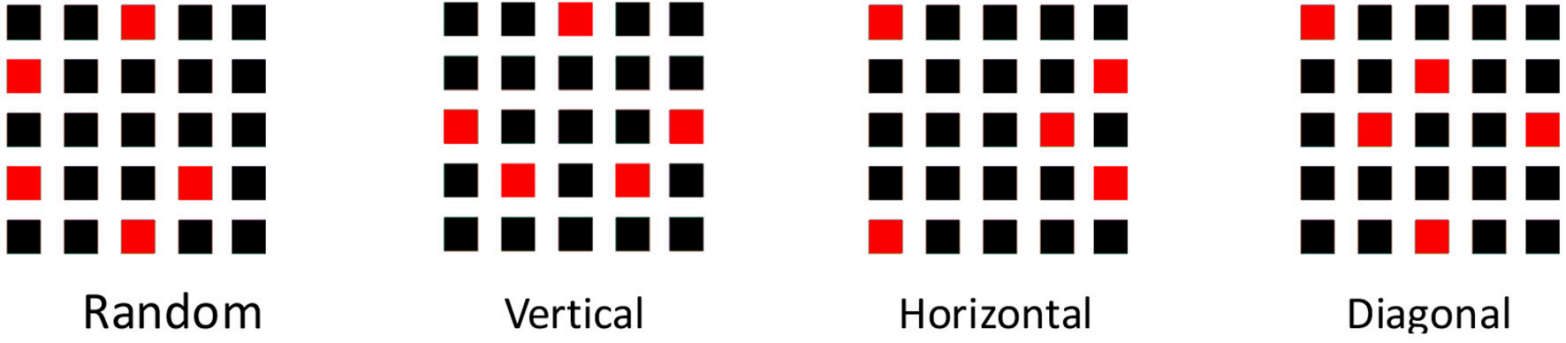
The matrix symmetry task stimuli at level 5 are displayed; random array, vertical symmetry, horizontal symmetry and diagonal symmetry conditions.

The Matrix Symmetry Task procedure was derived from the task stimulus arrays conventionally employed by Rossi-Arnaud et al. (Rossi-Arnaud et al., 2006; Pieroni et al., 2011). Examples of the arrays are shown in Figure 1 below.

The tasks were carried out in either group circumstances with adequate spacing between the young adult participants, or in single participant contexts with the older adult sample. In both contexts the task procedures were carried out under the supervision of the researchers. For all participants, task variant order was randomly allocated in a block-wise manner. In a given trial, the stimulus array was simultaneously presented on a screen for 3 s, and 2 s after the presentation the participants either identified the array configuration by pointing to blocks on a 5 × 5 wooden block array and recorded by the researcher (Rome), or by pointing to and marking cells on an A4 sheet with a blank array of 5 × 5 cells outlined (Newcastle). Participants were allowed to change their mind before confirming their response. After an initial practice of three trials at span level 1 (one red square), the ascending span procedure advanced with the progression criterion of two fully correct at each level with 3 trials per level (This was also the case with progression from the practice level). Thus, the task commenced from Level 1, one red square, on the 5 × 5 black cell array. Span was taken as the maximum level at which two fully correct responses were achieved. Figure 1 shows examples of the symmetry formats at Level 5. Feedback was not given on trial performance.

#### Analyses

The mean span data were analyzed using a 2 (age group) × 4 (symmetry) mixed factorial Analysis of Variance (ANOVA). *Post-hoc* tests were Bonferroni-corrected.

### Results

**Figure 2:**
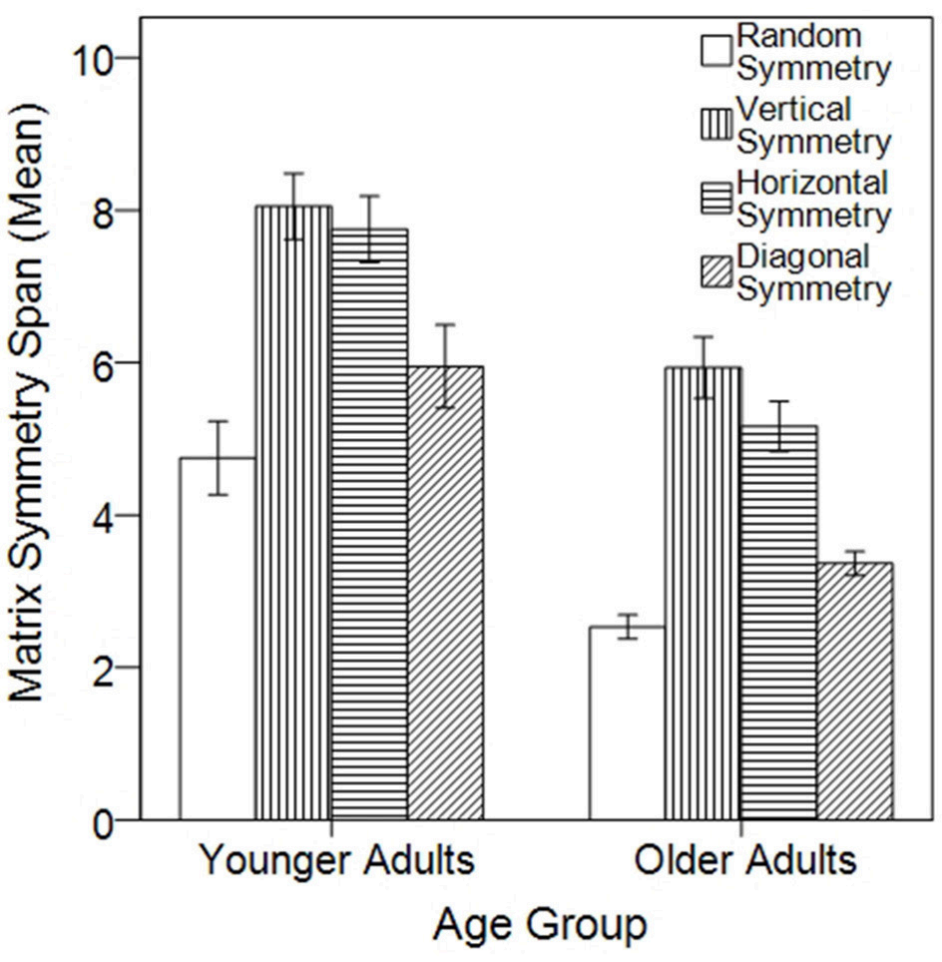
The effect of symmetry on matrix span as a function of age group. The mean (± 1 SE) matrix symmetry span performance is shown across the four symmetry conditions and the two age groups.

The data are displayed in Figure 2, which illustrates that older participants had numerically lower matrix span scores across all symmetry task conditions, relative to the younger adult age group. Indeed, the ANOVA revealed a significant effect of age group, *F*_(1, 48)_ = 37.75, *p* < 0.001, η_*p*_^2^ = 0.44, with means (and *SE*s) of 6.63 (0.30) and 4.25 (0.24) for the younger and older adult groups, respectively. There was also a significant effect of symmetry condition, *F*_(3, 46)_ = 37.56, *p* < 0.001, η_*p*_^2^ = 0.71 (*M*_RANDOM_ = 3.64, *SE* = 0.22; *M*_VERTICAL_ = 6.99, *SE* = 0.30; *M*_HORIZONTAL_ = 6.46, *SE* = 0.27; and *M*_DIAGONAL_ = 4.66, *SE* = 0.24). Importantly, there was no significant interaction between age group and symmetry condition, *F*_(3, 46)_ = 0.47, *p* = 0.71, η_*p*_^2^ = 0.03. *Post-hoc* analysis with Bonferroni correction revealed that performance in the random symmetry condition was significantly lower than the three symmetry conditions (all *p* ≤ 0.001). The vertical symmetry condition performance was significantly greater than the diagonal condition (*p* < 0.001), but not different from the horizontal condition (*p* = 0.114). Performance in the horizontal condition was significantly greater than the diagonal condition (*p* < 0.001). The lack of significant interaction effect, however, indicates that the older adult group was equally able to take advantage of the symmetry conditions.

Table 1A identifies the effect size (Cohen's *d, M*_1_–*M*_2_*/pooled standard deviation*) associated with these age differences. It is clear that there are large effect sizes associated with each individual task condition. Table 1B also shows that the effect sizes of the performance advantages for each symmetry condition are particularly large, with older adults consistently demonstrating a mean effect size of ~0.4 above the young adult group.

**Table 1:** Matrix symmetry task effect sizes associated with age group and task manipulation.

	**Effect size**
**A. EFFECT OF AGE IN EACH STIMULUS CONDITION**	
Random matrices	*d* = 1.356
Vertical symmetry matrices	*d* = 1.021
Horizontal symmetry matrices	*d* = 1.377
Diagonal symmetry matrices	*d* = 1.413
**B. EFFECTS Of SYMMETRY CONDITION**	
Younger adults	
Random vs. vertical symmetry	*d* = 1.615
Random vs. horizontal symmetry	*d* = 1.464
Random vs. diagonal symmetry	*d* = 0.522
Older adults	
Random vs. vertical symmetry	*d* = 2.026
Random vs. horizontal symmetry	*d* = 1.870
Random vs. diagonal symmetry	*d* = 0.982

Thus, despite a general decline in matrix symmetry task performance, there is no evidence for a decline in the ability to take advantage of physical complexity, at least in the form of symmetry, in the older adult group.

### Discussion

The results of Experiment 1 show a clear effect of age on matrix symmetry task performance (e.g., Logie and Maylor, 2009; Johnson et al., 2010; Brown et al., 2012). However, in terms of task performance across different symmetry conditions, there was improvement in all symmetry conditions, with large effect sizes for all of these in both age groups. Thus, although it was predicted that there may be a weaker advantage in performance for older adults when the task array condition was symmetrical, this was not supported. The older adult group was as effective as the younger adult group in taking advantage of symmetry in the array pattern, across the different forms of symmetry investigated. Even though they began from a relatively impaired baseline performance level in the control condition, they received as much benefit from all forms of symmetry, as compared with the younger adults. Furthermore, the effect sizes associated with these advantages in the older adult group were all very large. Thus, despite a decline in individual task performance, the older adults were able to effectively take advantage of symmetry in the memory array patterns. Therefore, the use of low-level physical properties of the VPT stimuli, in this case symmetry, does not seem to offer an explanation for the mechanisms underlying the deleterious effect of age on task performance.

## Experiment 2

In Experiment 2, younger and older adults again carried out a visual matrix task, however this time the matrix sets had been constructed either to constrain or enhance semantic affordance (see also Brown et al., 2006; Orme, 2009; Riby and Orme, 2013; Orme et al., 2017). Previous research suggests that older adults are impaired in accessing and retrieving LTM semantic content to support visual matrix recall (e.g., Burke and Light, 1981; Craik and Byrd, 1982; Healey and Kahana, 2016). Also, incorporating semantics in this visual working memory task appears to come at a cognitive cost (Brown and Wesley, 2013). Thus, it was predicted that younger adults would be more effective than older adults in taking advantage of the semantic affordance provided by the high semantic matrices set.

### Methods

#### Design

The experiment took the form of a cross sectional mixed factorial 2 × 2 design, and investigated the effects of adult age group (younger, older) and semantic affordance (low, high; repeated measures) on VWM capacity, as measured by the span level achieved in each task condition.

#### Participants

In total, 70 participants were recruited. A young adult group (*n* = 40) was opportunistically drawn from the Department of Psychology, Northumbria University. This group had a mean age of 19.5 (*SD* = 1.06 years; min = 19, max = 24), 32 of whom were female. The older age group comprised the same 30 older participants described in Experiment 1. All subjects gave written informed consent in accordance with the Declaration of Helsinki. Ethics permission was granted by the ethics committees of the Departments of Psychology at Northumbria University. No remuneration was given to participants and participation was voluntary, with the right to withdraw at any point in the procedure emphasized to participants.

#### Materials and procedure

##### Visual matrix task

**Figure 3:**
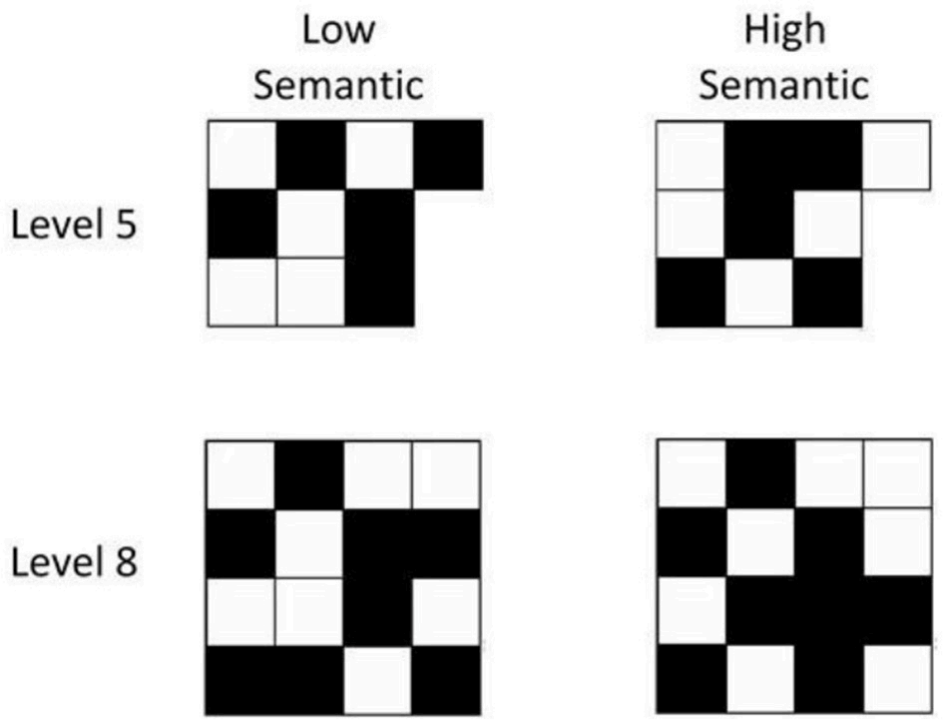
Examples of low and high semantic visual matrix stimuli at levels 5 and 8.

Orme (2009) asked participants to indicate how much of the visual matrix pattern to which they felt they could apply meaning, on a scale of 1 (none of the pattern) to 7 (all of the pattern; see also Brown et al., 2006). This was defined as all or parts of the pattern resembling “familiar objects or symbols,” or where they recognized shapes or configurations which could be difficult to explicitly name. From an initial set of over 1,000 matrix stimuli, Orme constructed two sets of visual matrix stimuli systematically varying in their semantic affordance. Examples of the stimuli are shown in Figure 3. All stimuli within a given level possessed an equal number of black and white cells.

The general procedure was similar to the Experiment 1 general protocol, where the tasks were carried out in either group circumstances with adequate spacing between the young adult participants, or in single participant contexts with the older adult sample. In both contexts the task procedures were carried out under the supervision of the researchers. For all participants, task variant order was randomly allocated. For a given trial, the stimulus was presented for 3 s on a monitor. After a maintenance interval of 1 s, the participant indicated their recall of the black cell locations by touching a cell on the blank visual matrix pattern on the screen, which turned the white cell black. Participants were allowed to change their decision, by touching the same cell again. After practice trials at Levels 2 and 3 the participants progressed through the ascending span protocol, with a progression criterion of minimally 1 correct out of the 3 trials at each level including the practice levels (Della Sala et al., 1997, 1999; Brown et al., 2006). Span was taken as the maximum level at which 1 correct response was achieved. Figure 3 identifies examples at level 5 and Level 8, note that as the Level increases there is a commensurate increase in array size. Feedback was not given on trial performance.

#### Analyses

The mean span data were initially analyzed using a 2 (age group) × 2 (semantic affordance) mixed factorial Analysis of Variance (ANOVA). *Post-hoc* tests were Bonferroni-corrected.

#### Results

**Figure 4:**
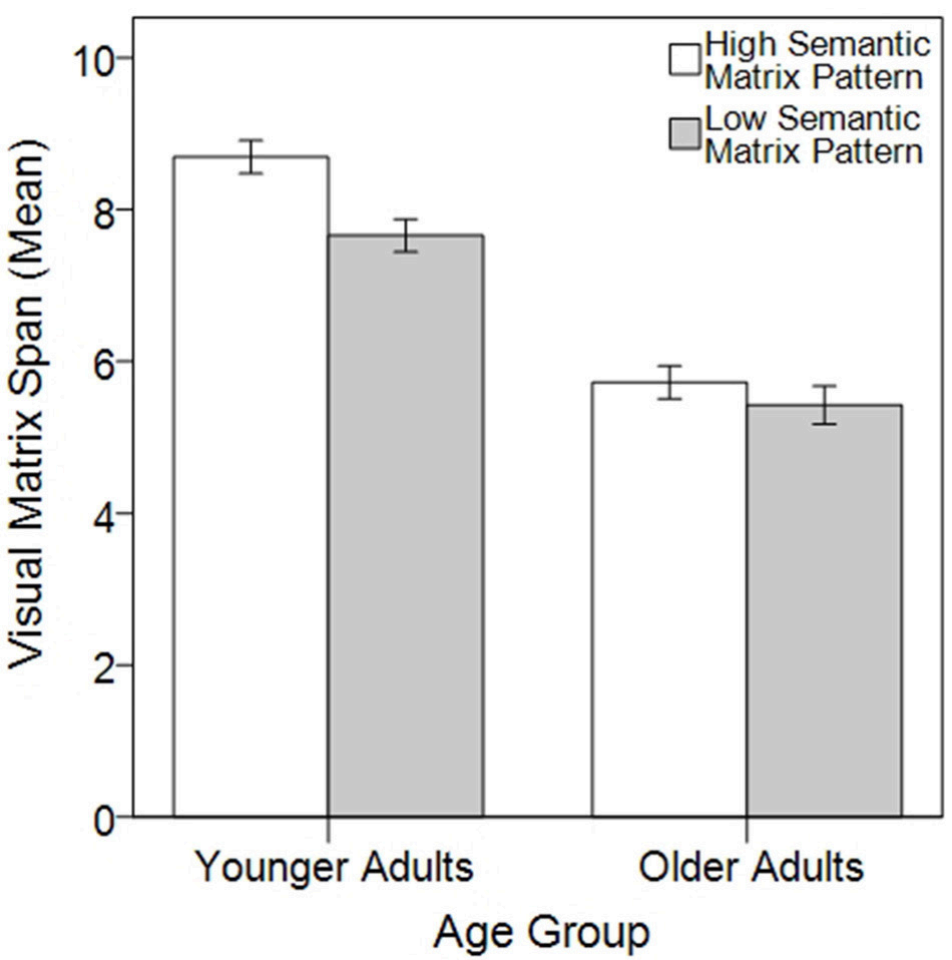
The effect of semantic affordance on matrix span as a function of age. The mean (± 1 SE) visual matrix span performance is shown across the two semantic affordance conditions and the two age groups.

The data are displayed in Figure 4 below, which shows a decrease in visual matrix performance in older adult participants, relative to the younger adult age group, across both semantic conditions. However, while younger adults appear to improve from low to high semantic affordance, older adults do not appear to do so. Indeed, the ANOVA revealed a significant effect of age group, *F*_(1, 68)_ = 81.57, *p* < 0.001, η_*p*_^2^ = 0.55, in which younger adults outperformed older adults, with means (and *SE*s) of 8.18 (0.19) and 5.58 (0.22), respectively. In addition, there was a significant effect of semantic affordance, *F*_(1, 68)_ = 21.28, *p* < 0.001, η_*p*_^2^ = 0.24, with means (and *SE*s) of 7.21 (0.16) and 6.54 (0.17), respectively, for the high and low semantic conditions. Importantly however, there was a significant interaction between age group and semantic affordance, *F*_(1, 68)_ = 6.48, *p* = 0.013, η_*p*_^2^ = 0.09 (see Figure 4).

*Post-hoc* simple effects analysis indicated that young adults significantly differed in their performance across the two semantic affordance conditions, *t*_(39)_ = 5.14, *p* < 0.001, (*M*_HIGH_ = 8.69, *SE* = 0.21; *M*_LOW_ = 7.66; *SE* = 0.22). However, the older adult group showed no significant difference between the two conditions, *t*_(29)_ = 1.51, *p* = 0.143 (*M*_HIGH_ = 5.72, *SE* = 0.24; *M*_LOW_ = 5.43, *SE* = 0.25). Table 2A below also identifies the effect sizes (Cohen's *d*) associated with these age differences. It is clear that there is a particularly large effect size associated with age in the high semantic affordance condition. Figure 4 and Table 2B below indicate that the significant interaction effect results from the relative lack of semantic benefit in the older adult group.

**Table 2:** Visual matrix task effect sizes associated with age group and task manipulation.

	**Effect size**
**A. YOUNG VS. OLDER AGE**	
Visual matrix high semantic span	*d* = 2.317
Visual matrix low semantic span	*d* = 1.629
**B. LOW VS. HIGH SEMANTIC STIMULI**	
Younger adults	*d* = 0.754
Older adults	*d* = 0.233

#### Discussion

The results of Experiment 2 indicated that there was a very large age associated difference in baseline visual matrix task performance (Bruyer and Scailquin, 1999; Beigneux et al., 2007; Logie and Maylor, 2009; Johnson et al., 2010; Brown et al., 2012), and that this existed across both semantic affordance conditions. However, as indicated by the interaction effect, and as shown in Figure 4, it was also apparent that improvement in the high semantic condition was only present in the younger adult age group. This contrast was also evident in the difference in effect sizes presented in Table 2. Thus, only the younger adult age group was able to take advantage of the high semantic affordance set of matrice stimuli. Previous research suggested that the benefit of semantic affordance comes at a cognitive cost, specifically upon domain-general executive resources in working memory (Brown and Wesley, 2013). The present study provides novel evidence that older adults appear less able to engage the cognitive processes required in order to gain a benefit of the availability of LTM-based semantics, in the context of this age-sensitive visual working memory task.

## General discussion

Visual Working Memory tasks such as the VPT evidence age-associated deficits that are amongst the largest observed in the memory literature (Logie and Maylor, 2009; Johnson et al., 2010). What is not so clear is the explicit identification of the cognitive processes which underlie such a large change. Within a conceptualization of VWM task performance in terms of generic executive attentional resources combined with domain-specific activations (e.g., Logie, 2011; Baddeley, 2012; Li et al., 2014; Shipstead and Yonehiro, 2016; Swanson, 2017), this research focused upon two factors which could contribute to the observed age effects. The first factor was associated with physical complexity (Sun et al., 2011), and to what extent older adults could take advantage of the reduced complexity present in symmetrical pattern arrays (Rossi-Arnaud et al., 2006, 2012). Second, we investigated the extent to which older adults were able to take advantage of enhanced semantic affordance opportunities in the pattern arrays (Hamilton et al., 2003; Ricker et al., 2010; Brown and Wesley, 2013). This second approach reflected the notion of perceived complexity (Sun et al., 2011) and the importance of LTM access, retrieval and scaffolding of abstract, “novel” matrix patterns.

First, the results indicated very large effect sizes associated with age, and as such replicate the Logie and Maylor (2009; also Johnson et al., 2010) adult lifespan data. Interestingly, there was significant variability in the effect sizes associated with age, with vertical symmetry matrix pattern performance evidencing an effect size less than half that observed with the visual matrix high semantic task, for example. Overall, though, the findings support the strong effect of age on VWM that has previously been observed (e.g., Swanson, 2017). However, the most important aim for the present research was to determine the extent to which older and younger adults differed in their use of perceptual and semantic affordance within the matrix pattern stimuli.

Regarding perceptual affordance, the older adult group were no less efficient in utilizing the perceptual cues of pattern symmetry in order to improve their performance in the matrix symmetry task. The effect sizes associated with these advantages were particularly large in the older participant group. Thus, even though symmetry perception has previously exhibited a small decline in performance (Herbert et al., 2002) the older adult group were able to effectively use this information and maintain less physically complex pattern representations in VWM. This makes sense when interpreted in the context of recent findings that, at the neural level, VWM representations are noisier, or less distinctive, with age (e.g., Grady, 1996; Park et al., 2004; Spreng et al., 2010). Less physically complex patterns may therefore help to alleviate the problem. What is unclear, however, is whether the benefit is relatively automatic, or cost-free, in terms of cognitive resources, or whether older adults are explicitly using the symmetry to aid recall (i.e., top-down rather than bottom-up). This question would be a useful avenue for future research.

In contrast, it is clear that in the context of taking advantage of the semantics within the visual matrix patterns, there was a reliable difference between the two age groups. This was indicated primarily by the significant interaction between age group and semantic affordance in which the older adult group did not significantly enhance their visual matrix performance in the high semantic condition, while the younger adults were able to do so (see also Brown et al., 2006; Brown and Wesley, 2013; Riby and Orme, 2013; Orme et al., 2017). Additionally, the effect size associated with semantic affordance was much smaller in the older age group. Thus, unlike the younger adults, we infer that the older age group was unable to access, retrieve, and/or associate pertinent LTM semantics in order to scaffold their VWM performance. This could have arisen from a decrease in the executive attentional control needed to retrieve from LTM (Unsworth et al., 2014). Unsworth et al. (2014) built upon earlier work (Unsworth et al., 2010) in differentiating the contribution of generic attentional control (as measured by 3 tasks requiring varying inhibitory control) from the more specific attentional control needed to access and retrieve from LTM. However, the function and target of the retrieval process does differ in the protocol in the present study from that employed by Unsworth and colleagues. In order to assess efficacy in the LTM retrieval process, Unsworth et al. (2010, 2014) presented stimuli for later recall from secondary memory, e.g., paired associates lists, delayed free recall lists, immediate free recall measures of non-recency items. Thus, in all of these protocols the participant is accessing and retrieving from a *recent* partially activated secondary memory or LTM. In the visual matrices task, as currently administered, the participant either has to associate automatically activated semantic representations, or actively search for *pre-existing* LTM semantic information, which can both give meaning and support to the “novel” visual pattern (Brown and Wesley, 2013). Healey and Kahana (2016, p. 30) refer to this as the “…*rich ensemble of activated representations …*” (see also Verhaeghen et al., 2006). However, whether the semantics were automatically or strategically activated, the benefit of semantics appears to draw upon central executive resources (Brown and Wesley, 2013), either for binding the semantics and novel representations together, or for developing or switching between strategies. Challenged executive attentional resources may therefore underlie the findings currently observed with the semantic version of the task (Phillips and Hamilton, 2001; Braver and West, 2008). Furthermore, even within younger adults, reported strategy use varies markedly (Brown and Wesley, 2013). Thus, future research could usefully investigate the strategies spontaneously used across the two age groups, as this is likely to impact the performance levels achieved.

Another mechanism which may underlie the apparent difficulty for older adults effectively to use semantics is processing speed (Salthouse, 1996). Previous evidence using the modified Visual Patterns Test (Brown et al., 2006), which limits meaning and verbal coding, showed that processing speed was the greatest predictor of performance in older adults (Brown et al., 2012). This could reflect limitations in the speed of encoding and/or rehearsal, but could also be implicated in the ability to identify and/or actively bind semantic and novel representations. Indeed, recent research has identified that processing speed is implicated in age effects in visual short-term memory, specifically in more complex (multiple object) visual arrays (Guest et al., 2015). In the present context, even if visual semantics can be activated relatively automatically at encoding (Logie, 2011; Brown and Wesley, 2013), age-related slowing could reduce the efficiency with which those representations are activated and/or enter the VWM system. However, it is important to note that, in Brown et al. (2012), although processing speed was the strongest predictor of performance, central executive capacity, specifically when working with visuo-spatial material (i.e., “visuo-spatial organization”), was also uniquely predictive of VWM performance. Notably, this was not the case for executive attention ability, as measured with a verbal-based task (verbal fluency). Thus, visuo-spatial organization was specifically implicated, and could be related to strategy selection and implementation, such as drawing upon visual semantics. This supports our argument above, that executive attentional capacity may be implicated in the current pattern of findings. Thus, it is possible that both executive attentional functioning and processing speed make significant contributions to visual working memory performance in older age (Salthouse, 1996; Salthouse et al., 2003; Brown et al., 2012). Future research could consider the extent to which attentional resources, for example in the form of strategy selection and implementation, or verbal recoding, is the challenge for the older adult group in this visual matrix task, or whether processing speed can account for the lack of semantic recruitment. Manipulation of the encoding and maintenance durations would perhaps enable the processing speed account to be assessed.

The relatively small age-associated effect sizes of the low semantic versus the high semantic task performance is also of interest. This suggests that in visual memory protocols which are less demanding of executive attention, age associated change may be smaller (Phillips and Hamilton, 2001). This is evidenced in the findings of Peich et al. (2013), who investigated adult age associated change within task performance when the protocol requiring fine detailed representation of either single or multiple visual stimulus arrays. The participants were required to remember either the color or orientation of the stimuli. This qualitative, representational visual memory task is less likely to draw upon the executive attention control processes discussed immediately above. The authors found significant age associated changes, but of a much smaller order than that observed in the current visual matrix high semantic condition, certainly when considering recall of the visual properties of single stimulus arrays.

One should note that although there is strong evidence in these current findings that the two visual working memory tasks make qualitatively different demands upon the broader functional architecture of working memory it is possible that in older adult group there was the possibility of some transfer of learning across the two tasks. In addition, without a detailed knowledge of the cognitive profile of the older adult group, there may be some constraint in identifying the precise age related effects.

In conclusion, the aim of the research was to identify, through experimental manipulation, the impact of varying semantic and perceptual opportunities upon the scaffolding of visual working memory task performance. The results indicated that the older adult group were less effective at utilizing semantic opportunities to improve and scaffold performance of a visual matrix working memory task. This could, at least in part, be due to some generic constraint in executive attentional resources. Challenges to a specific attentional control process; accessing and retrieving pertinent information from LTM, is one such candidate. In contrast, the older adult age group demonstrated evidence of being effective in making use of perceptual cues and the redundancy afforded by symmetry in visual arrays. However, whether the symmetry was actively used by the older adults to scaffold VWM performance, or whether the benefit was more automatic, remains to be seen. Thus, within the same group of older adult participants, experimental manipulations of the memory array format led to systematic differences in the strength of the age-associated mnemonic differences. Importantly, the effects presently observed were all in the context of spontaneous task performance with these particular stimulus comparisons. Future work could therefore usefully address how these factors affect older adults' performance under different task instructions or with other stimulus variants.

## Ethics statement

This experiment was carried out in accordance with the recommendations of procedures and protocols approved by the Ethics Committee of Northumbria and La Sapienza Universities with written informed consent from all participants.

## Author contributions

CH substantial contributions to the conception or design of the work, acquisition, analysis, and interpretation of data for the work. Drafting the work and final approval of the version to be published and agreement to be accountable for all aspects of the work in ensuring that questions related to the accuracy or integrity of any part of the work are appropriately investigated and resolved. LB substantial contributions to analysis, and interpretation of data for the work. Drafting the work and final approval of the version to be published and agreement to be accountable for all aspects of the work in ensuring that questions related to the accuracy or integrity of any part of the work are appropriately investigated and resolved. CR-A Substantial contributions to the conception or design of the work, acquisition, analysis, and interpretation of data for the work. Drafting the work and final approval of the version to be published and agreement to be accountable for all aspects of the work in ensuring that questions related to the accuracy or integrity of any part of the work are appropriately investigated and resolved.

### Conflict of interest statement

The authors declare that the research was conducted in the absence of any commercial or financial relationships that could be construed as a potential conflict of interest.
